# Effect of endocrine disruptors on bacterial virulence

**DOI:** 10.3389/fcimb.2023.1292233

**Published:** 2023-11-06

**Authors:** Audrey Thiroux, Jean-Marc Berjeaud, Romain Villéger, Alexandre Crépin

**Affiliations:** Université de Poitiers, UMR CNRS 7267, Ecologie et Biologie des Interactions, Poitiers, France

**Keywords:** endocrine disruptors, virulence, pathogens, antibiotic tolerance, biofilm

## Abstract

For several decades, questions have been raised about the effects of endocrine disruptors (ED) on environment and health. In humans, EDs interferes with hormones that are responsible for the maintenance of homeostasis, reproduction and development and therefore can cause developmental, metabolic and reproductive disorders. Because of their ubiquity in the environment, EDs can adversely impact microbial communities and pathogens virulence. At a time when bacterial resistance is inevitably emerging, it is necessary to understand the effects of EDs on the behavior of pathogenic bacteria and to identify the resulting mechanisms. Increasing studies have shown that exposure to environmental EDs can affect bacteria physiology. This review aims to highlight current knowledge of the effect of EDs on the virulence of human bacterial pathogens and discuss the future directions to investigate bacteria/EDs interaction. Given the data presented here, extended studies are required to understand the mechanisms by which EDs could modulate bacterial phenotypes in order to understand the health risks.

## Introduction

Endocrine disruptors (EDs) are substances that are able to disrupt the endocrine system by altering several biological functions of the body such as development, metabolism, nervous and reproductive system (Schug et al., 2011). EDs have structures mimicking endogenous steroid hormones, including estradiol (E2) or androgen. They interfere with hormone synthesis or receptor binding by altering the hormone homeostasis of the endocrine system (Yue et al., 2023). Among the major chemical substances considered as EDs and found in numerous everyday products, there are bisphenols and phthalates used in plasticizers and parabens and triclosan used as preservatives in cosmetics, food and beverage packaging, toys, carpet and pesticides. Their extensive use participates in the release, dissemination and accumulation of chemical substances in the environment. Indeed, these compounds are mostly introduced into the environment via water networks. For these reasons, EDs are now recognized as serious public health concern, potentially emerging as one of the leading environmental risks.

If the effects of EDs on human health have been previously reported, the possible effect of these compounds on bacterial communities have been poorly described. In this context, the concept of “microbial endocrinology” first described by Lyte and collaborators is relevant (Lyte and Ernst, 1992). Indeed, authors suggested that inter-kingdom signaling supports the idea of interaction between endogenous substances (hormones) of the host and microorganisms (Lyte et al., 1996; Lyte, 2014; Lyte et al., 2018; Boukerb et al., 2021). Some hormones such as catecholamines affect the physiology and behavior (production of toxins, adhesins, motility, *quorum sensing*, biofilm formation) of various human pathogens (*Escherichia coli, Pseudomonas aeruginosa, Acinetobacter baumannii*). Thus, the reported effects of hormones on bacteria have raised questions about the presence of bacterial sensors for these hormones. Indeed, bacteria use two-component systems to sense environmental signals and induce signal transduction resulting in the regulation of virulence gene expression. The two-component system consists of a membrane-bound histidine kinase (HK) that detects environmental changes (hormones, xenobiotics, nutrients) and autophosphorylates at the histidine residue using an ATP molecule. Phosphorylation of HK is transferred to the response regulator by phosphorylation of its aspartate residue. The activated intracellular response regulator enables the transduction of an appropriate signal, generally the induction or repression of one or more sets of genes involved in complex pathways (Shaw et al., 2022). For example, it has been demonstrated that *E. coli* QseC kinase receptor is able to detect both QS auto-inducing molecules (AI-3) but also eukaryote neurotransmitters (epinephrine and norepinephrine) and thus adapt its behavior by modulating genes expression involved in motility (*flhDC* promoter) (Clarke et al., 2006). In addition, 10 µM epinephrine has been shown to promote the pathogenicity of *Pseudomonas aeruginosa* H103 on *Galleria mellonella* model, as well as increased adhesion and biofilm formation (Cambronel et al., 2019). Another interesting fact, catecholamines can also promote bacteria growth in *P. aeruginosa* (Freestone et al., 2012), modulate sensitivity and resistance to antibiotics in *A. baumannii* (Inaba et al., 2016), stimulate horizontal gene transfer between enteric bacteria (Peterson et al., 2011), increase resistance to oxidative stress by positively regulating the expression of the superoxide dismutase (*sodA*) gene in *S. enterica* serovar Typhi (Karavolos et al., 2008). Hormone sensing by pathogens is not limited to catecholamines. Indeed, it has been demonstrated that the C-type natriuretic peptide (CNP) is detected by *P. aeruginosa* via a specific protein named AmiC which is an orthologue of the human natriuretic peptide receptor (Rosay et al., 2015).Therefore, since EDs are analogues to hormones, it seems essential to evaluate the risk of their exposure on the behavior and physiology of pathogenic bacteria.

Relationships between the presence of EDs and their effect on microbial communities have been previously described. Few reports have highlighted that EDs in various abundances and forms could disturb the gut microbiota composition (dysbiosis) with consequent pathological outcomes in animals, including humans (Hampl and Stárka, 2020). However, data regarding the direct effect of EDs on the virulence of pathogenic bacteria are still missing and exposure to these substances could constitute an additional parameter in the appearance and development of microbial infections that has to be evaluated.

Thus, the present review aims to summarize the current knowledge on the effect of EDs on bacterial pathogens and the consequences on their virulence. The **Table 1** summarizes the data published to date on the effect of these substances on bacterial virulence.

**Table 1:** Overview of the effects of endocrine disruptors on bacterial virulence reported in the literature.

Endocrine Disruptors	Strain	Methods	Main results	References
**Bisphenols**	*Streptococcus mutans*	Exposure of planktonic bacteria to 50 µg/mL of Bisphenol A glycidyl methacrylate (bis-GMA)	• Bis-GMA increases sugar transport, intracellular polysaccharide accumulation and resistance to hydrogen peroxide	(Kim et al., 2019)
	*Escherichia coli* DH5ɑ *Escherichia coli* HB101 or *Salmonella enterica*	0.1, 1.0, 10.0 and 100 µg/L bisphenol S (BPS) and bisphenol AF (BPAF)	• BPS and BPAF exposure increase conjugative transfers • No effect of BPS and BPAF on growth and cell membrane permeability • BPS and BPAF can modulated expression of global regulator genes, vertically transferred genes, mating pair formation gene, DNA-transfer-and-replication gene, outer membrane protein genes and stress-related response genes	(Feng et al., 2022)
	*Pseudomonas aeruginosa* H103	Exposure to 1 nM, 1 µM, 10 µM and 100 µM BPA during biofilm formation	• Exposure to 1 nM BPA increased biofilm formation	(Thiroux et al., 2023)
**Parabens**	*Acinetobacter calcoaceticus and Stenotrophomonas maltophilia* *(*opportunistic and emerging pathogen)	Exposure to Methylparaben (MP), Propylparaben (PP) and butylparaben (BP) at 150 ng/L or the mixture of the three parabens during 7 and 26 days.	• Modulation of culturality and bacterial density from a mono-species biofilm as a function of long-term paraben exposure • After 26 days of exposure to MP, cell density of *A. calcoaceticus* biofilms increased by 85% • Biofilm thickness increased (44%) with MP on *S. maltophilia* with polypropylene (PPL) • MP increased swimming motility (+141%), gelatinase (+41%) and protease (+73%) production	(Pereira et al., 2023)
	*Pseudomonas aeruginosa* H103	Exposure to 1 nM, 1 µM, 10 µM and 100 µM EP and MP during biofilm formation, motility experiment, or infection of A549 cells	• Exposure to 1 nM EP increased biofilm formation • Increased adhesion to A549 lung epithelial cells when exposed to 1 nM EP • Swarming motility increased with MP at 1 nM, 10 μM and 100 μM	(Thiroux et al., 2023)
**Phthalates**	*Helicobacter pylori* ATCC 43054	Co-exposure of human gastric epithelial cells (AGS) to 2-ethylhexyl-phthalate at 80 μM (DEHP) and *H. pylory* at MOI of 100: 1	• Co exposure of *H. pylori and* DEHP increased cytotoxicity and gastric epithelial cell apoptosis	(Lin et al., 2013)
	*Pseudomonas aeruginosa* H103 and clinical strains isolates	Exposure to 10^-3^ to 10^-10^ M of DEHP, DINP (bis (7-methyloctyl) phthalate), DEP (diethyl phthalate) and DBP (dibutyl phthalate). DEHT (bis (2-ethylhexyl) terephthalate), ATBC (tributyl acetyl citrate), TXIB (2,2,4-trimethyl-1,3-pentanediol diisobutyrate, DOIP (di-2-ethylhexyl isophthalate) and DIOP (dicyclohexylphthalate)	• No effect of Phthalates/their substitutes on the growth of *P. aeruginosa* • No effect of phthalates or/their substitutes on pyocyanin and pyoverdine production • Phthalates/their substitutes increase biofilm formation and pellicle formation • DEHP, DINP, DIOP and DOIP (10^-3^ M) increased membrane fluidity • Morphological alterations at 10^-3^ M DBP	(Louis et al., 2022)
	*Pseudomonas aeruginosa* ATCC 15629	Exposure to DMP, DnHP and DEHP at 1 to 5 μg/L	• DMP, DnHP and DEHP promotes *P. aeruginosa* biofilm formation and resistance to free chlorine • Increased expression of the genes involved in QS, extracellular polymeric substances excretion and oxidative stress resistance	(Wang et al., 2022)
**Triclosan (TCS)**	*Pseudomonas aeruginosa* H103	Exposure to 1 nM, 1 µM, 10 µM and 100 µM DBP during biofilm formation	• 1 nM DBP increased biofilm formation	(Thiroux et al., 2023)
	*Staphylococcus aureus*	Bacteria exposed to 50 nM TCS. Attachment assay on human serum, collagen, fibronectin keratin and glass surfaces) Rats gavaged with TCS (100 mg/kg/day), nasally inoculated with a small inoculum (10^5^ CFU) or a large inoculum (10^8^ CFU) *of S. aureus* SH1000	• Correlation between level TCS and carriages rates of *S. aureus* in human nasal secretions: <175 nM – 32-27% against >176 nM -64% • Increased attachment of *S. aureus* attachment to host proteins and glass surface when exposed to TCS • TCS exposure promotes colonization by *S. aureus* in an *in vivo* model of nasal colonization and more susceptibility to colonization with small inoculum	(Syed et al., 2014)
	*Streptococcus mutans* ATCC 25175 *Streptococcus mutans* ATCC 35668	Exposure to TCS at ½ MIC, ¼ MIC during biofilm formation Adhesion assay on human gingival epithelial cell line OBA-9 of *S. mutans* cultures exposed to TCS (1/2, 1/4 or 1/8 MIC) Gene expression *atlA*, *gtfC*, *gtfB comD luxS*, assessed by RT-qPCR after 2h exposure to TCS (1/2, 1/4 or 1/8 MIC)	• TCS increased biofilm formation in *S. mutans* • At ½ MIC and ¼ MIC TCS, the biofilm is thicker with aggregates and microcolonies • At ½ MIC (42.5%) and ¼ MIC TCS, adhesion of ATCC 25175 is enhanced on OBA-9. No significative effect on cell surface hydrophobicity • Increased expression of gene involved adhesion and biofilm at TCS sub inhibitory concentrations	(Bedran et al., 2014)
	*Escherichia coli* K-12 *LE392* (donor plasmid RP4), *Escherichia coli* K-12 MG1655 and *Pseudomonas putida* KT2440 (recipients)	TCS exposure at 0.02, 0.2, 2, 20, 200 and 2000 μg/L	• TCS (2 µg/L) increased horizontal transfer frequency of plasmid RP4 by 6.2-fold (intra-genera) • Increased frequency of conjugative transfer associated with overproduction of ROS or increased cell membrane permeability. Increased gene expression involved in metabolism with high TCS level	(Lu et al., 2018b)
	*Escherichia coli* DH5ɑ plasmid pUC19	TCS exposure at 0.02, 0.2, 2, 20, 200 and 2000 μg/L	• TCS promoted uptake efficacy of exogenous DNA in strains recipient *E. coli* • TCS at 0.02 μg/L and 2000 μg/L increased ROS production and promote transformation • Change in membrane properties observed at high concentration of TCS • Up-regulation of secretion systems Sec (*secA, secB* and *secY*) system Tat (*tatA* and *tatB*) up-regulated following TCS exposure	(Lu et al., 2020)
	*Escherichia coli* K12	TCS exposure at 0, 0.02, 0.2 and 2 mg/L during 30 days with everyday change fresh, liquid LB with respective concentrations of TCS Antibiotics: amoxicillin (AMX), ampicillin (AMP), cephalexin (LEX), chloramphenicol (CHL), kanamycin (KAN), levofloxacin (LVX), norfloxacin (NOR) and tetracycline (TET)	• TCS increased of mutation frequency for several antibiotics at 0.2 mg/L • Chronic exposure to TCS induces stable genetic changes within generations, induces risk-resistant mutants and develops resistance to oxidative stress	(Lu et al., 2018a)
	Uropathogenic *Escherichia coli* (UPEC)	UPEC clinical isolates long-term exposed to TCS at 5×MBC in agar plates and repeated for 12 passages	• Induction of cross resistance by TCS to nitrofurantoin and ciprofloxacin • TCS reduced pathogenicity in 5/8 isolates tested on *G. mellonella* model	(Henly et al., 2019)
	*Escherichia coli* MG1655 *Staphylococcus. aureus* FPR3575 *Escherichia coli* UTI89	TCS (200 ng/mL) exposure 30 min prior to the addition of antibiotics in *E. coli* MG1655: Ampicillin (100 µg/mL), Streptomycin (50 µg/mL), Kanamycin (50 µg/mL), Ciprofloxacin (100 ng/mL) TCS (100 ng/mL) exposure 30 min with addition 1,000 ng/ml ciprofloxacin on *E. coli* UTI89 Female wild-type C3H/HeN mice, exposed to TCS (1000 ng/mL) during 21 days. Infection with on *E. coli* UTI89. Treatment 24h with ciprofloxacin (25 mg/kg).	• Protective effect of TCS on *E. coli* MG1655 against antibiotics at concentrations that are normally lethal • TCS-mediated tolerance requires ppGpp • TCS pre-treatment resulted in *E. coli* UTI89 being 10 times more tolerant to ciprofloxacin *in vitro* test • TCS reduces the efficacy of ciprofloxacin *in vivo* test (mice) • Bacterial load > 100 times higher in urine (P < 0.0001) and > 10 times higher (P < 0.0001) in the bladder of TCS-treated mice compared to control animals	(Westfall et al., 2019)
	*Pseudomonas aeruginosa* PA01	Exposure to TCS (100 µM) and tobramycine (400 or 500 µM) TO-PRO™-3 iodide dye for permeabilization test Determination of pump efflux activity by accumulation of ethidium bromide and determination of intracellular accumulation and extrusion of conjugated tobramycin with the fluorescent dye Texas Red (TbTR) Change membrane potential (Δψ) by staining with fluorescent indicator Δψ DiOC2(3)/flow cytometry Murine wound model SKH-1	• TCS combined with tobramycin causes synergistic permeabilization of cells within biofilms • TCS reduces the activity of efflux pumps, leading to increased accumulation of TbTR from mature biofilm • By acting as a protonophore, TCS inhibits tobramycin induced the membrane potential (Δψ) • TCS and tobramycin show enhanced efficacy in an *in vivo* murine *w*ound model	(Maiden and Waters, 2020)
	*Streptococcus mutans* UA159 ATCC 700610	TCS- Methacrylate (TM) monomer, which was developed and incorporated into an experimental resin composite (TEGDMA) Cell metabolism–XTT metabolic assay Biofilm characteristic analysis (CLSM) Live/Dead Quantitative virulence gene expression	• Decreased cellular metabolism of *S. mutans* on the TM-containing composite, compared with the TM-free composite • Decrease in biovolume, average thickness, roughness and surface values of the biofilm formed on the composite containing TM compared to that which does not contain it • the contact time (4 h or 24 h) between TM-composite and *S. mutans* down-regulated the *gbpB* and *covR* and up-regulated the *gtfC* gene expression	(de Souza Araújo et al., 2018)
	*Staphylococcus aureus* ATCC 6538	WT bacteria (the passage 0 [P0] strain), those passaged 10 times on TCS (strain P10) and those passaged a further 10 times on TCS-free (strain x10) TCS MIC/MBC determination (final concentrations, 232 μg/ml to 0.23 μg/ml) Biofilm characteristic analysis (CLSM) Live/Dead Determination of hemolysin, DNase and coagulase activity. *G. mellonella* pathogenesis assay	• TCS exposure selects for isolates with reduced TCS susceptibility • Variability of colonies formed by the TCS-adapted (P10) strain markedly smaller and more heterogeneous than those formed by parent strain (P0) • TCS-adapted *S. aureus* (P10) grows more slowly than P0 in planktonic and biofilm modes • Thinner biofilms when exposed (P10) or having been exposed (x10) to TCS compared to P0 • The *in vitro* hemolytic, DNase and coagulase activity of TCS-adapted *S. aureus* (P10) is lower than that of P0 • P10 and x10 are less virulent in *G. mellonella*	(Latimer et al., 2012)

### Bisphenols

Bisphenol A (BPA) and analogues (bisphenol AF, BPAF and bisphenol S, BPS) are synthetic substances mainly used in the production of polycarbonate plastics and as a feedstock for epoxy resins. The maximum quantified BPA concentrations found in drinking water and source waters from North America, Europe and Asia were 0.099 μg/L 0.014 μg/L and 0.317 μg/L, respectively (Arnold et al., 2013). Unfortunately, high concentrations of bisphenols are found in India surface water with mean concentration of 1.39 µg/L and 302 ng/g from a honey sample (bisphenol S; BPS) (Yamazaki et al., 2015; Česen et al., 2016). In France, BPA has been banned since 2012 in all childcare products (REACH). A 2016 study has shown that exposure to bisphenol A leads to changes in the composition of the intestinal microbiota in a mouse model, suggesting a potential effect on bacteria physiology (Javurek et al., 2016).

In humans, BPA is rapidly metabolised in the liver by glucuronidation (BPA-GA) and excreted in the bile. However, the gut microbiota hosts certain families of enterobacteria such as *Escherichia*, *Salmonella*, *Klebsiella*, *Shigella* and *Yersinia* pathobionts, which are able to produce enzymes known as intestinal beta-glucuronidases. These enzymes destroy the glucuronic acid sugars bound to xenobiotic compounds (such as ED) (Sakamoto et al., 2002). As a result, BPA is deconjugated (free form), favouring continued exposure to EDs in the host and in opportunistic pathogens, which could contribute to changes in microbial composition and favour enteric pathogen colonization (Little et al., 2018).

Bisphenol A glycidyl methacrylate (bis-GMA) has been demonstrated to inhibit the growth of planktonic cultures of *Streptococcus mutans* and a decrease of the bacteria viability. Nevertheless, the bis-GMA has been reported to contribute to the intracellular accumulation of polysaccharides that could favor the adhesion of the bacteria to surfaces and consequently could promote the formation of biofilms (Kim et al., 2019). In 2022, Feng et al., have demonstrated that bisphenol S (BPS) and bisphenol AF (BPAF) (0.1-100.0 μg/L) could increase the frequency of conjugative antibiotic resistance gene transfer between *E. coli* DH5α carrying the RP4 plasmid and recipient strains *E. coli* HB101 (2-5 fold) or *Salmonella enterica* (4-5 fold). In addition, authors reported a downregulation of global regulator gene system (Grg) and an upregulation of Mating pair formation system (Mpf) and DNA transfer replication genes (Dtr) system, without significant damage on cell permeability and stress related response (Feng et al., 2022). In 2023, our team showed that BPA exposure promotes *P. aeruginosa* H103 biofilm formation at a concentration of 1 nM, a trace concentration found in French aquatic resources (Thiroux et al., 2023).

### Parabens

Parabens are esters of 4-hydroxybenzoic acid commonly used as preservatives in personal care products, in medicines and secondarily in food (Soni et al., 2005). They are generally of synthetic origin but can be produced naturally (fruit). World production is estimated to average 8,000 tons per year, with 1,000 to 10,000 tons per year for methyl-paraben (MP), 100 to 1,000 tons per year for ethyl-paraben (EP) and propyl-paraben (PP) (REACH). High concentrations of parabens are found in surface waters, in wastewater as well as in the human population (Wei et al., 2021). Indeed, 0.120 - 0.182 µg/L of MP were found in drinking water in Spain (Valcárcel et al., 2018) and 0.002 - 0.0082 µg/L of EP were found in surface water in China regarding indoor environment, 50 ng/g - 26.2 µg/g MP, 9 ng/g - 1.06 µg/g EP, 70 ng/g -11.15 µg/g PP, 6 ng/g -0.86 µg/g butylparaben (BP), 2 - 27 ng/g benzylparaben (BzP) were found in indoor dust (Chen et al., 2018). In human, 17.6-27.437 µg/L MP were found in hair (Karzi et al., 2019) and 15.4 µg/L MP, 4.76 µg/L EP, 7.82 µg/L PP, 0.4 µg/L BP are found in urine (China) (Zhang et al., 2020).

In 2023, Peirera et al., found that chronic exposure for 7 and 26 days to parabens alone or in cocktail (MP, PP, BP at 0.15 µg/L or the mixtures of the three parabens) modulated the expression of virulence factors of two pathogens, *Acinetobacter calcoaceticus* and *Stenotrophomonas maltophilia*. Indeed, an increase of biofilm formation was observed, the increase in swimming motility (141%), in the production of gelatinase and protease, 41 and 73% respectively, were observed in the presence of MP (Pereira et al., 2023). More recently, our team demonstrated that 1 nM EP increased biofilm formation *of P. aeruginosa* H103 and also enhanced adhesion to pulmonary epithelial cells. In contrast, swim motility was reduced in the presence of EP (1 nM; 1 µM; 10 µM and 100 µM) (Thiroux et al., 2023). EP appeared to be a substance that could promote adhesion, biofilm formation at the expense of motility which could facilitate colonization of the pathogen in water networks and in humans.

### Phthalates

Phthalates are mainly used in the plastic and coating industries. Indeed, diethylhexyl phthalate (DEHP), dibutyl phthalate (DBP), diethyl phthalate (DEP), di-isononyl phthalate (DiNP) and di-iso-decyl phthalate (DiDP) are used as plasticizers to produce polyvinyl chloride (PVC). Dimethyl phthalate (DMP) and DEP, also known as short-branched low molecular weight phthalates, are used to make personal care products (e.g., hair products), pharmaceuticals and medical devices. Unfortunately, they are easily released into the environment because phthalates do not form a covalent bond with plastic material. Huge freights of plastic materials end-up in environmental water and soil where they persist for long times. Human exposure to phthalates leached from plastic into water but also food and other products applied directly to the human body, is almost unavoidable (Wang and Qian, 2021).

In China, the mean concentrations of nine phthalate esters detected in water, soil and sediment samples were 4.11 µg/L, 408 ng/g and 1200 ng/g, respectively. DBP was predominant in water (50.6%) and DEHP predominated in soil (69.6%) and sediment (83.1%) (Zhu et al., 2022). Furthermore, indoor air contamination was predominant for DEHP with 40.6% (Kashyap and Agarwal, 2018). In France, the French National Agency of Sanitary Safety (ANSES) campaign performed between 2013-2014, emphasize DEP as the predominant compound in surface water with a maximum concentration of 0.41 µg/L. In contrast, DBP is the ED the most frequently detected in treated tap water samples with a maximum concentration of 1.3 µg/L. Surprisingly, DEHP which is often identified in the environment, was only detected in a single surface water sample at 0.81 µg/L (RAPPORT AST révisé de l’Anses relatif à la Campagne nationale de mesures de l’occurrence de composés émergents dans les eaux destinées à la consommation humaine acides haloacétiques – chlorates – phtalates - Rouxiella chamberiensis, campagne 2016-2017, 2020).

If phthalates are ubiquitously found in aquatic resources and soil, interaction with native microbes has been previously reported and degradation by microorganisms is considered as the most effective means of their elimination from the environment (Kumar et al., 2017; Shariati et al., 2023). Indeed, authors demonstrated an ability of *Pseudomonas* sp. V21b to degrade the phthalate DBP by producing metabolic intermediates such as phthalate monobutyl. For their assay, they used a minimal medium supplemented with DBP (1994 µg/L) as the only carbon source, they initially added 1994 µg/L of DBP. After 192 h, the residual amount was only 857 µg/L suggesting that 57% of DBP was degraded suggesting. Thus, this environmental strain could therefore be useful for bioremediation purposes. In addition, a study has enabled the biochemical characterization of the inducible component of phthalate isomer dioxygenases from an environmental isolate of *P. aeruginosa* (PP4) (Karandikar et al., 2015).

In 2013, Lin et al. have reported the possible effect of DEHP exposure on the consequences of infection by *Helicobacter pylori*, a bacterium that could lead to gastroduodenal ulcers and gastric cancer. Their work showed that co-exposure to DEHP and *H. pylori* promotes cytotoxicity of the bacterium and increases apoptosis of gastric epithelial cells. Indeed, viability tests on gastric epithelial cells (AGS gastric adenocarcinoma) revealed that about 87% of the cells were dead after 18h of exposure with DEHP (80µM) and *H. pylori* (MOI 100) compared with just over 50% with *H pylori* alone. Factors involved in apoptosis were measured by Western blot from AGS cells, in particular the Bcl-2/Bax ratio, as well as the expression of caspases 3 and 8. The co-exposure of *H. pylori* and DEHP at 80 µM disturbed the balance of the Bcl2/Bax ratio and the expression of caspase 3 was significantly increased. Therefore, these results suggested that the co-exposure of DEHP and *H. pylori* promoted the pathogenesis of the bacterium on the one hand and on the other hand, could enhance cancer development (Lin et al., 2013). However, the mechanisms behind have not been described. In order to explore the mechanisms of interaction between bacteria and theses EDs, few studies have investigated the possible effect of phthalates on bacterial physiology. Recently, our group demonstrated that exposure to DMP, di-n-hexyl phthalate (DnHP) and DEHP promotes biofilm formation by *Pseudomonas aeruginosa* PAO1 (ATCC 15629) at concentrations between 1 and 10 µg/L. Wang et al. (2022) have also reported that phthalate exposure promoted *Pseudomonas* biofilm formation and resistance to free chlorine (Wang et al., 2022). Authors demonstrated that three phthalates DMP, DnHP and DEHP induced an increase of polysaccharidic matrix (30.3-82.3%) and eDNA (10.3-39.3%) production as well as antioxidative defenses such as superoxide dismutase and catalase. Similarly, 2,2,4-trimethyl-1,3-pentanediol diisobutyrate (TXIB), a phthalate substitute used at 10^-3^ M increased biofilm formation (2.8 fold) in *P. aeruginosa* H103 (Louis et al., 2022). Moreover, we have shown that exposure of *P. aeruginosa* to DBP at a concentration found in French aquatic resources (1 nM) can significantly increase biofilm formation (Thiroux et al., 2023).

*P. aeruginosa* is a human opportunistic pathogen and a major health issue in hospitals, being responsible for nosocomial infections (Qin et al., 2022), especially in resuscitation units or for immunocompromised patients. Increased biofilm ability when exposed to low concentrations of EDs in water could not even promote *P. aeruginosa* biofilm development in water networks, but possibly in humans too. Based on these data, the ability of phthalates to modulate physiologic changes in pathogenic bacteria that promote their virulence could become a critical health issue that has almost not been investigated. Further research is required to highlight the possible risk of bacterial exposure to phthalates for animals, including humans, in order to prevent and treat bacterial infections.

### Triclosan

Triclosan (TCS), also known under the following names; “Irgasan^®^”, “Lexol 300”, “Cloxifenolum”, is a biocide commonly used since the 70s. The antibacterial property lies in its ability to inhibit bacterial synthesis of type II fatty acids (FasII) by inactivation of the enzyme enoyl-acyl reductase (FabI). It is mainly present in care and cosmetic articles (85%) (soaps, toothpastes, mouthwashes), 10% in plastics and polymers and 5% in textile processing (clothing, bedding products). In the United States, soaps containing TCS were banned since September 2016 by the Food and Drug Administration (Brose et al., 2019). As for the European Union, the regulation imposes a maximum concentration of 0.3% of TCS for cosmetic products and 0.2% for mouthwash solutions. The Florence statement published in the journal Environmental Health Perspectives in 2017 describes the major concerns of the scientific community regarding the health risks of TCS (Halden et al., 2017). Indeed, studies showed the persistent effect of the molecule (Halden and Paull, 2005) and its bioaccumulation in the environment (Dhillon et al., 2015). TCS is found ubiquitously (environment, human), in surface water (nM concentrations range) (Weatherly and Gosse, 2017), in indoor dust (20 ng/g-3.27 µg/g) (Chen et al., 2018), in plasma (0.16-127.6 µg/L) and in urine (0.16-11909 µg/L) (Geens et al., 2009; Geer et al., 2017).

Few studies have investigated the effect of TCS on bacterial virulence. Work by Syed et al. (2014) showed that the presence of TCS seems to be associated with *Staphylococcus aureus* colonization in the nasal cavity (Syed et al., 2014). *S. aureus* is a major bacterial human pathogen and the causative agent of multiple human infections. The bacteria binding ability to host proteins (collagen, fibronectin, keratin, human serum) is an additional asset in the colonization process. Attachment tests of *S. aureus* to human proteins in the presence of TCS (50 nM) have shown a higher attachment rate compared to non-exposed *S. aureus* (Syed et al., 2014). Thus, the presence of TCS could be an advantage in the development of infections, but the mechanisms have to be deciphered. During the same year, Bedran et al. (2014) focused on *Streptococcus mutans*, an agent causing dental plaque infection (carries). It was shown that ½ and ¼ MIC concentrations of TCS, promote biofilm formation by 6.2 and 5 times, respectively (Bedran et al., 2014). In addition, the ability to adhere to gingival epithelial cells was increased by 42.5% (at ½ MIC). Finally, the genes involved in adhesion and biofilm formation (*atlA, comD, gtfB, gtfC, luxS*) were quantified by RT-qPCR. The expression levels of these genes were mostly upregulated for a ½ MIC, with a 3.6-, 3.1- and 4-fold increase in *gtfC, comD* and *luxS* expression, respectively (Bedran et al., 2014). However, a study also performed on *S. mutans* indicates that the TCS-Methacrylate (TM) monomer, incorporated into an experimental composite resin (TEGDMA) is able to decrease cell viability, reduce the biovolume, the average thickness, the coefficient of biofilm roughness and decrease the expression of virulence genes (*gbpB* and *covR*) (de Souza Araújo et al., 2018). Therefore, the use of a composite containing TM would disrupt the viability and potentially the establishment of a functional biofilm. The use of such monomer seems to be a relevant antimicrobial in preventive and restorative dentistry. Although these studies indicate cate contradictory data on the effect of TCS exposure on *S. mutans* virulence, it is important to report that the experimental exposures involve different modes of TCS exposure either by the addition of the substance at sub-inhibitory concentrations (Bedran et al., 2014) or by the use of a composite provided with TCS (de Souza Araújo et al., 2018). Therefore, it would seem that the way in which the pathogen is exposed to TCS could modulate its virulence.

In some bacterial species, such as *S. aureus*, the presence of TCS at sublethal concentrations results in the development of small colony variant (SCV) (Bazaid et al., 2018) which are slow growing subpopulations that may be recovered from patients with persisting infections. Paradoxically *S. aureus* adaptation to sub-inhibitory concentrations of TCS led to a decrease in hemolytic, DNase and coagulase activities when compared to the parental strain (Latimer et al., 2012). Moreover, the pathogenicity measured by the mortality rate of *G. mellonella* indicates that the strain adapted to TCS exposure is less virulent (Latimer et al., 2012). In 2018, Lu et al. reported that exposure to environmental concentrations (0.02 µg/L to 2000 µg/L) of TCS could promote multidrug resistance gene conjugative transfer in bacteria (Lu et al., 2018b). Indeed, TCS exposure promoted generation of reactive oxygen species (ROS) in three tested microorganisms. Indeed an increase of ROS was obtained at 2 μg/L TCS for the donor *E. coli* K-12 LE392, while for recipients *E. coli* K-12 MG1655 and *P. putida* KT2440 strains the generation of ROS was obtained at 20 and 2000 μg/L TCS, respectively (Lu et al., 2018b). In addition, an increase in membrane permeability was observed at 20 µg/L for the donor strain and 200 µg/L for recipient strain *P. putida* KT2440 (Lu et al., 2018b). In contrast, no significant change has been observed whatever the TCS concentration used for the recipient *E. coli* K-12 MG1655 (Lu et al., 2018b). Moreover, an increased expression of genes associated with the SOS response (*umuC, dinB* and *dinD*) for donor strain *E. coli* K-12 LE392 have been obtained during exposure to low TCS concentrations (0.02 μg/L) (Lu et al., 2018b). With the ROS scavenger added, the increase of ROS generation and conjugative transfer caused by TCS were significantly inhibited, indicating that TCS-induced ROS generation promotes the conjugative transfer of plasmid RP4 from *E. coli* K-12 LE392 in both the recipient strains *E. coli* K-12 MG1655 and *P. putida* KT2440 (Lu et al., 2018b). This work demonstrated that TCS, at relevant environmental concentrations, could promote both intra-generic conjugative transfer and inter-generic transfer of genetic elements (plasmids) and thus contributes to the emerging problem of antibiotic resistance through the acquisition of antibiotic resistance genes (Lu et al., 2018b). In addition, a 30-day exposure of *E. coli* K-12 to TCS induces genetic mutations (*fabI, frdD, marR, acrR* and *soxR*), as well as overexpression of the gene coding for beta-lactamase and multi-drug efflux pumps (Lu et al., 2018a). In 2020, the same team also demonstrated that TCS could increase frequency of transformation, associated with an increase of ROS production and membrane permeability (Lu et al., 2020). Moreover, the Sec secretion system and the type IV pilus secretion system were increased in the presence of TCS, promoting the acquisition of extracellular DNA (plasmid pUC19) by the competent model (*E. coli* DH5ɑ) (Lu et al., 2020). Exposure to TCS, also found in catheterized medical devices, can induce changes in clinical strains of uropathogenic *E. coli* (UPEC). Indeed, a reduction of the sensitivity to biocide as well as to antibiotics has been observed following repeated exposure to triclosan, at the minimum inhibitory concentrations (MIC), minimum bactericidal concentrations (MBCs) and the minimum biofilm eradication concentrations (MBEC) determined for all isolates. The relative pathogenicity of UPEC isolates was studied in a *G. mellonella* model and showed a significant decrease (5/8 isolates) in death rate after long-term exposure to TCS. This work indicates the adaptation of clinical isolates to biocides and highlights the significance of the selection of an anti-infective catheter-coating agent (Henly et al., 2019).

Westfall et al. (2019) have shown that TCS pretreatment appears to provide protection to *E. coli* against antibiotic concentrations normally lethal to the bacteria. Indeed, the survival of the bacteria pretreated with TCS was multiplied by 1,000 and 10,000 times in the presence of kanamycin (50 µg/mL) and ciprofloxacin (0.1 µg/mL) respectively. The frequency of persistent cells was increased in the presence of TCS and associated to an increase in the synthesis of alarmone guanosine tetraphosphate (ppGpp) (Westfall et al., 2019). In addition, experiments conducted on an uropathogenic clinical isolate*, E. coli* UTI89, indicated that exposure of the pathogen to TCS increases its tolerance to ciprofloxacin (1 µg/mL) by up to 10 times. In an animal model, mice exposed to water-containing TCS (100 µg/mL) showed a reduced response to ciprofloxacin treatment during infection with the UPEC strain UTI89. Bacterial titers were >100-fold higher in the urine (P < 0.0001) and >10-fold higher (P < 0.0001) in the bladders of TCS-treated mice versus control animals (Westfall et al., 2019). If authors detected two putative metabolized forms of TCS in the urine of exposed mice, the mechanisms of TCS on the bacteria are still unclear. Taken together, data regarding pathogens exposure to TCS highlight deleterious “side effects” of this ubiquitous ED, mostly involved in increased biofilm formation and antibiotic tolerance. Extended studies are today required to highlight the mechanisms involved, including long-term experiments with chronic exposure to TCS at concentrations found in consumer products.

Nevertheless, Maiden et al. (2020) showed that TCS (100 μM) deplete the membrane potential of the growing *P. aeruginosa* biofilm, resulting in decreased efflux pump activity (Maiden and Waters, 2020). This disruption leads to increased intracellular accumulation of tobramycin (500 µM) and high antimicrobial activity *in vitro* in a mouse wound model. The protonophore activity of TCS is due to the presence of the hydroxyl group. Indeed, experiments using an analog of TCS, methyl-TCS, showed no effect on membrane potential, on the activity of efflux pumps or even on cell membrane permeabilization alone or in combination with tobramycin (Maiden and Waters, 2020). Although contradictory effects between the protective effect of TCS (Westfall et al., 2019) versus the synergistic effect of TCS coupled with tobramycin (Maiden and Waters, 2020), it seems to be that only antibiotics that corrupt translation (aminoglycosides and tetracycline) act synergistically or are enhanced when combined with TCS.

## Discussion

The ubiquitous of substances considered as endocrine disruptors in soil, sediments, surface water, drinking water and human biological matrix is a major public health issue. The effects of EDs on microbial communities, as a modulator of microbiota composition and bacterial virulence, have been demonstrated. Data from the literature indicate that EDs are able to modulate the behavior of pathogenic bacteria. Physiological responses such as phenotypic changes and increased expression of virulence factors have been observed. To date, in order to anticipate the threats posed by the exposure of pathogenic bacteria to EDs, extensive studies are required.

**Figure 1:**
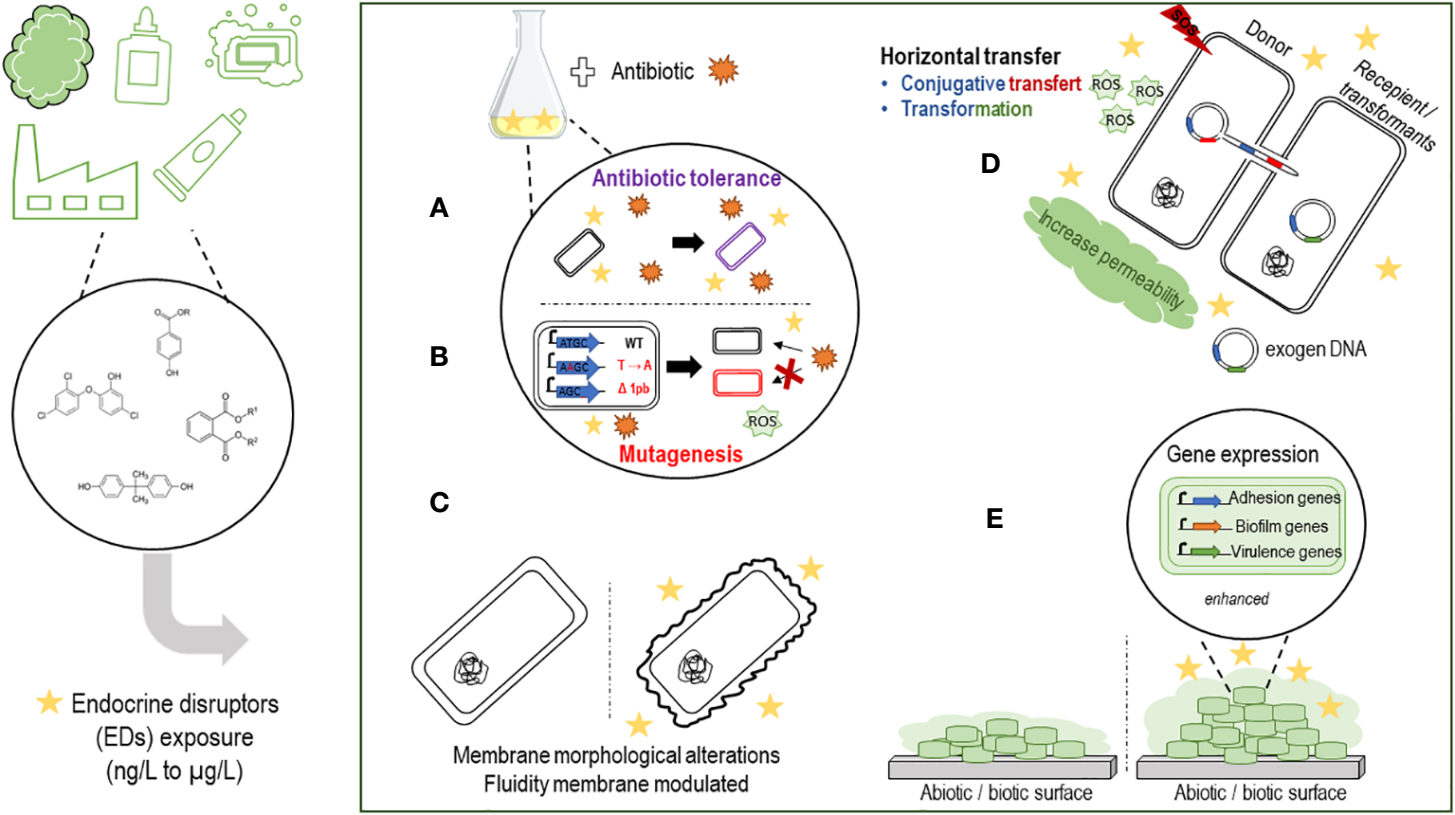
Schematic summarizing the effects of endocrine disruptors on the virulence of pathogenic bacteria. **(A)** EDs pretreatment seems to protect bacteria against antibiotics, with selection of a persistent bacterial population. **(B)** Chronic exposure to EDs seems to increase the frequency of mutations (deletion, substitution, polymorphism) contributing to an increase in antibiotic resistance genes. **(C)** Exposure to EDs can induce morphological changes or phenotypic changes. **(D)** EDs could promote and accelerate the spread of resistance genes against antibiotics. Horizontal transfers such as transformation or conjugation seem to be enhanced in the presence of EDs associated with an SOS response, an increase of ROS (reactive oxygen species) production with an increase or not of membrane permeability, depending on the ED considered. **(E)** Adhesion, biofilm formation and virulence gene expression can be promoted by the presence of EDs on both abiotic and biotic surfaces.

In the course of this work, we found that exposure to EDs prior to antibiotic treatment could result in high levels of antibiotic tolerance. TCS pre-treatment of *E. coli* (MG1655) is able to induce tolerance to several antibiotics (ampicillin, streptomycin, kanamycin and ciprofloxacin), depending on *ppGpp* synthesis (**Figure 1A**). TCS exposure also increased the frequency of persistent bacteria. These observations were confirmed *in vivo* in a mouse model exposed to TCS and infected with *E. coli* (UTI89), responsible for urinary tract infections (Westfall et al., 2019). Chronic exposure to TCS induced resistance in *E. coli* K12 to several antibiotics (levofloxacin, amoxicillin, chloramphenicol, tetracycline). In addition, mutation frequencies and the generation of ROS were increased. Genomic analysis identified genetic changes following exposure to TCS in six antibiotic-resistant mutants. Among the mutants sequenced, insertion, substitution and frameshift mutations were identified. Exposure to EDs and TCS in particular, induces mutagenic effects, which contribute to antibiotic resistance mechanisms, by modulating gene expression levels (overexpression of the gene encoding beta-lactamase; efflux pump system, AcrAB-TolC) (**Figure 1B**). Overall, ED exposure appears to enhance the acquisition of exogenous material, contributing to the spread of antibiotic resistance and accentuating potential therapeutic failures in the hospital environment. Alterations in membrane morphology and fluidity, via SEM analysis and anisotropy measurement respectively, were observed during exposure to phthalates (10^-3^ M DBP) on the pathogen *P. aeruginosa* (Louis et al., 2022) (**Figure 1C**).

ED exposure, such as bisphenols and TCS, promotes horizontal transfers of multidrug resistance genes via transformation (Lu et al., 2020) and/or conjugation mechanisms (Lu et al., 2018b) between inter/intra-generic strains (*E. coli, S. enterica, P. putida*). Increased membrane permeability as well as increased ROS expression have been observed in mechanisms involving conjugative antibiotic resistance gene transfers and appear to be essential for promoting the transformation mechanism (Lu et al., 2020) (**Figure 1D**). However, membrane disruption has not been observed in all studies (Feng et al., 2022), so the promotion of horizontal transfer may depend on the nature of the disrupting substance.

Other studies including ours have shown that EDs can increase biofilm formation by *P. aeruginosa*, *S. mutans, A. calcoaceticus* and *S. maltophilia* when exposed to phthalates (Louis et al., 2022; Wang et al., 2022; Thiroux et al., 2023), to TCS (Bedran et al., 2014), or to parabens (Pereira et al., 2023; Thiroux et al., 2023), increase adhesion to biotic/abiotic surfaces (Bedran et al., 2014; Syed et al., 2014; Thiroux et al., 2023) for *S. aureus* and *S. mutans* and modulate the virulence of P. *aeruginosa*, *A. calcoaceticus* and *S. maltophilia* in mice models (Henly et al., 2019; Westfall et al., 2019) (**Figure 1E**). Based on our experience and on literature data, we suppose that certain ED, such as EP, could promote adhesion, biofilm formation at the expense of motility in a *quorum sensing*-dependant manner, which in turn could facilitate colonization of the pathogen in water networks and in humans. However, extensive *in vivo* studies are required to address this hypothesis.

The mechanisms of genetic transfer material seem to be reinforced during exposure to certain EDs, which raises questions about the consequences in terms of dispersion and dissemination of resistant bacteria. RNAseq experiments during chronic exposure to EDs (alone or combination) would be relevant to address the mechanisms behind the modulation of the pathogenic behavior through the regulation of gene expression. Moreover, docking analysis could allow identification of ED sensors in the bacteria. Indeed, an orthologous receptor of human C-type natriuretic peptide has been identified in *P. aeruginosa* (PA14), named AmiC (Rosay et al., 2015). Studies by us and others on *P. aeruginosa* raise the question of the role of bacterial factors involved in *quorum sensing* in the bacteria response to ED, but extended studies are required. Mechanistic/molecular knowledge could be an advantage to better understand and anticipate the risks of interaction between EDs and the pathogen, notably by limiting the access of the EDs to bacterial sensors and thus reduce the consequences on virulence. Despite the measures taken by governments to restrict the use of suspected or proven EDs, these molecules are to date ubiquitous into the environment, mainly in water resources. Although substitutes to EDs are proposed, such as tributyl acetylcitrate (ATBC), 2,2,4-trimethyl-1,3-pentanediol diisobutyrate (TXIB), bis-2-ethylhexylterephthalate (DEHT), diisononyl cyclohexane-1,2-dicarboxylate (DINCH) as phthalate substitutes (Substituts de phtalates dans les jouets pas de risque mis en évidence pour la santé des enfants de moins de trois an, 2016), a health risk assessment of the effect of these molecules on bacteria physiology is required.

Furthermore, because of the daily exposure to EDs found in food and water, we believe that all the effects observed with EDs on the virulence of pathogenic bacteria should be investigated to the microorganisms present in the gut microbiota. Indeed, the intestinal microbiota is composed not only of bacteria, but also of viruses (bacteriophages) and fungi that surround them. We therefore wondered whether the changes mentioned concerning pathogenic bacteria might also affect the interaction between bacteria and phages, leading to a modification in the composition of the intestinal virome (phageome). In the future, quantifying the relative abundances of phages in gut microbiota exposed or not to ED should be considered (Duan et al., 2022).

To conclude, several studies have reported that exposure to chemical substances classified as EDs have the potential to promote the virulence of bacterial pathogens. Through increased resistance to antibiotics, biofilm forming capacity and enhanced colonization abilities, exposure of pathogenic bacteria to EDs could pose additional threats to human health and should be carefully considered. To date, extensive investigations are required to evaluate the deleterious impact of ED exposure on bacteria-associated diseases.

## Author contributions

AT: Investigation, Writing – original draft, Writing – review & editing, Formal Analysis. J-MB: Project administration, Supervision, Validation, Writing – review & editing, Funding acquisition. RV: Supervision, Validation, Writing – review & editing. AC: Supervision, Writing – review & editing, Validation.

## References

[B1] Arnold S. M. Clark K. E. Staples C. A. Klecka G. M. Dimond S. S. Caspers N.. (2013). Relevance of drinking water as a source of human exposure to bisphenol A. J. Expo Sci. Environ. Epidemiol. 23 (2), 137–144. doi: 10.1038/jes.2012.66 22805988 PMC3580800

[B2] Bazaid A. S. Forbes S. Humphreys G. J. Ledder R. G. O’Cualain R. McBain A. J. (2018). Fatty acid supplementation reverses the small colony variant phenotype in triclosan-adapted staphylococcus aureus: genetic, proteomic and phenotypic analyses. Sci. Rep. 8 (1), 3876. doi: 10.1038/s41598-018-21925-6 29497096 PMC5832852

[B3] Bedran T. B. L. Grignon L. Spolidorio D. P. Grenier D. (2014). Subinhibitory concentrations of triclosan promote streptococcus mutans biofilm formation and adherence to oral epithelial cells. PloS One 9 (2):e89059. doi: 10.1371/journal.pone.0089059 24551218 PMC3923858

[B4] Boukerb A. M. Cambronel M. Rodrigues S. Mesguida O. Knowlton R. Feuilloley M. G. J.. (2021). Inter-kingdom signaling of stress hormones: sensing, transport and modulation of bacterial physiology. Front. Microbiol. 12, 690942. doi: 10.3389/fmicb.2021.690942 34690943 PMC8526972

[B5] Brose D. A. Kumar K. Liao A. Hundal L. S. Tian G. Cox A.. (2019). A reduction in triclosan and triclocarban in water resource recovery facilities’ influent, effluent, and biosolids following the U.S. Food and Drug Administration’s 2013 proposed rulemaking on antibacterial products. Water Environ. Res. 91 (8), 715–721. doi: 10.1002/wer.1101 30859670

[B6] Cambronel M. Tortuel D. Biaggini K. Maillot O. Taupin L. Ré́hel K.. (2019). Epinephrine affects motility, and increases adhesion, biofilm and virulence of Pseudomonas aeruginosa H103. Sci. Rep. 9 (1), 20203. doi: 10.1038/s41598-019-56666-7 31882963 PMC6934790

[B7] Česen M. Lambropoulou D. Laimou-Geraniou M. Kosjek T. Blaznik U. Heath. D.. (2016). Determination of bisphenols and related compounds in honey and their migration from selected food contact materials. J. Agric. Food Chem. 64 (46), 8866–8875. doi: 10.1021/acs.jafc.6b03924 27792318

[B8] Chen J. Hartmann E. M. Kline J. Van Den Wymelenberg K. Halden R. U. (2018). Assessment of human exposure to triclocarban, triclosan and five parabens in U.S. indoor dust using dispersive solid phase extraction followed by liquid chromatography tandem mass spectrometry. J. Hazard Mater 360, 623–630. doi: 10.1016/j.jhazmat.2018.08.014 30149349

[B9] Clarke M. B. Hughes D. T. Zhu C. Boedeker E. C. Sperandio V. (2006). The QseC sensor kinase: A bacterial adrenergic receptor. Proc. Natl. Acad. Sci. U.S.A. 103 (27), 10420–10425. doi: 10.1073/pnas.0604343103 16803956 PMC1482837

[B10] de Souza Araújo I. J. de Paula A. B. Bruschi Alonso R. C. Taparelli J. R. Innocentini Mei L. H. Stipp R. N.. (2018). A novel Triclosan Methacrylate-based composite reduces the virulence of Streptococcus mutans biofilm. PloS One 13 (4), e0195244. doi: 10.1371/journal.pone.0195244 29608622 PMC5880362

[B11] Dhillon G. Kaur S. Pulicharla R. Brar S. K. Cledón M. Verma M.. (2015). Triclosan: current status, occurrence, environmental risks and bioaccumulation potential. Int. J. Environ. Res. Public Health 12 (5), 5657–5684. doi: 10.3390/ijerph120505657 26006133 PMC4454990

[B12] Duan Y. Young R. Schnabl B. (2022). Bacteriophages and their potential for treatment of gastrointestinal diseases. Nat. Rev. Gastroenterol. Hepatol. 19 (2), 135–144. doi: 10.1038/s41575-021-00536-z 34782783 PMC8966578

[B13] Feng M. Ye C. Zhang S. Sharma V. K. Manoli K. Yu X. (2022). Bisphenols promote the conjugative transfer of antibiotic resistance genes without damaging cell membrane. Environ. Chem. Lett. 20 (3), 1553–1560. doi: 10.1007/s10311-022-01397-x

[B14] Freestone P. P. Hirst R. A. Sandrini S. M. Sharaff F. Fry H. Hyman S.. (2012). Pseudomonas aeruginosa-catecholamine inotrope interactions: a contributory factor in the development of ventilator-associated pneumonia? Chest 142 (5), 1200–1210. doi: 10.1378/chest.11-2614 22556319

[B15] Geens T. Roosens L. Neels H. Covaci A. (2009). Assessment of human exposure to Bisphenol-A, Triclosan and Tetrabromobisphenol-A through indoor dust intake in Belgium. Chemosphere 76 (6), 755–760. doi: 10.1016/j.chemosphere.2009.05.024 19535125

[B16] Geer L. A. Pycke B. F. G. Waxenbaum J. Sherer D. M. Abulafia O. Halden R. U. (2017). Association of birth outcomes with fetal exposure to parabens, triclosan and triclocarban in an immigrant population in Brooklyn, New York. J. Hazard Mater 323, 177–183. doi: 10.1016/j.jhazmat.2016.03.028 27156397 PMC5018415

[B17] Halden R. U. Lindeman A. E. Aiello A. E. Andrews D. Arnold W. A. Fair P.. (2017). The florence statement on triclosan and triclocarban. Environ. Health Perspect. 125 (6), 064501. doi: 10.1289/EHP1788 28632490 PMC5644973

[B18] Halden R. U. Paull D. H. (2005). Co-occurrence of triclocarban and triclosan in U.S. Water resources. Environ. Sci. Technol. 39 (6), 1420–1426. doi: 10.1021/es049071e 15819193

[B19] Hampl R. Stárka L. (2020). Endocrine disruptors and gut microbiome interactions. Physiol. Res. 69 (Suppl 2), S211–S223. doi: 10.33549/physiolres.934513 33094620 PMC8603731

[B20] Henly E. L. Dowling J. A. R. Maingay J. B. Lacey M. M. Smith T. J. Forbes S. (2019). Biocide exposure induces changes in susceptibility, pathogenicity, and biofilm formation in uropathogenic *Escherichia coli* . Antimicrob. Agents Chemother. 63 (3), e01892–e01818. doi: 10.1128/AAC.01892-18 30642923 PMC6395906

[B21] Inaba M. Matsuda N. Banno H. Jin W. Wachino J. I. Yamada K.. (2016). *In vitro* reduction of antibacterial activity of tigecycline against multidrug-resistant Acinetobacter baumannii with host stress hormone norepinephrine. Int. J. Antimicrob. Agents 48 (6), 680–689. doi: 10.1016/j.ijantimicag.2016.09.022 27842757

[B22] Javurek A. B. Spollen W. G. Johnson S. A. Bivens N. J. Bromert K. H. Givan S. A.. (2016). Effects of exposure to bisphenol A and ethinyl estradiol on the gut microbiota of parents and their offspring in a rodent model. Gut Microbes 7 (6), 471. doi: 10.1080/19490976.2016.1234657 27624382 PMC5103659

[B23] Karandikar R. Badri A. Phale P. S. (2015). Biochemical Characterization of Inducible “Reductase” Component of Benzoate Dioxygenase and Phthalate Isomer Dioxygenases from Pseudomonas aeruginosa strain PP4. Appl. Biochem. Biotechnol. 177 (2), 318–333. doi: 10.1007/s12010-015-1744-6 26201480

[B24] Karavolos M. Spencer H. Bulmer D. Thompson A. Winzer K. Williams P.. (2008). Adrenaline modulates the global transcriptional profile of Salmonella revealing a role in the antimicrobial peptide and oxidative stress resistance responses. BMC Genomics 9, 458. doi: 10.1186/1471-2164-9-458 18837991 PMC2576261

[B25] Karzi V. Tzatzarakis M. Katsikantami I. Stavroulaki A. Alegakis A. Vakonaki E.. (2019). Investigating exposure to endocrine disruptors via hair analysis of pregnant women. Environ. Res. 178, 108692. doi: 10.1016/j.envres.2019.108692 31520825

[B26] Kashyap D. Agarwal T. (2018). Concentration and factors affecting the distribution of phthalates in the air and dust: A global scenario. Sci. Total Environ. 635, 817–827. doi: 10.1016/j.scitotenv.2018.04.158 29710605

[B27] Kim K. An J. S. Lim B. S. Ahn S. J. (2019). Effect of bisphenol A glycol methacrylate on virulent properties of streptococcus mutans UA159. Caries Res. 53, 84–95. doi: 10.1159/000490197 29961075

[B28] Kumar V. Sharma N. Maitra S. S. (2017). Comparative study on the degradation of dibutyl phthalate by two newly isolated Pseudomonas sp. V21b and Comamonas sp. 51F. Biotechnol. Rep. 15, 1–10. doi: 10.1016/j.btre.2017.04.002 PMC5447571 28580302

[B29] Latimer J. Forbes S. McBain A. J. (2012). Attenuated Virulence and Biofilm Formation in Staphylococcus aureus following Sublethal Exposure to Triclosan. Antimicrob. Agents Chemother. 56 (6), 3092–3100. doi: 10.1128/AAC.05904-11 22430975 PMC3370732

[B30] Lin C. H. Wu C. Y. Kou H. S. Chen C. Y. Huang M. C. Hu H. M.. (2013). Effect of di(2-ethylhexyl)phthalate on helicobacter pylori-induced apoptosis in AGS cells. Gastroenterol. Res. Pract. 2013, 924769. doi: 10.1155/2013/924769 24454344 PMC3876891

[B31] Little M. S. Pellock S. J. Walton W. G. Tripathy A. Redinbo M. R. (2018). Structural basis for the regulation of β-glucuronidase expression by human gut Enterobacteriaceae. Proc. Natl. Acad. Sci. U.S.A. 115 (2), E152–E161. doi: 10.1073/pnas.1716241115 29269393 PMC5777068

[B32] Louis M. Tahrioui A. Verdon J. David A. Rodrigues S. Barreau M.. (2022). Effect of phthalates and their substitutes on the physiology of pseudomonas aeruginosa. Microorganisms 10 (9), 1788. doi: 10.3390/microorganisms10091788 36144390 PMC9502294

[B33] Lu J. Jin M. Nguyen S. H. Mao L. Li J. Coin L. J. M.. (2018a). Non-antibiotic antimicrobial triclosan induces multiple antibiotic resistance through genetic mutation. Environ. Int. 118, 257–265. doi: 10.1016/j.envint.2018.06.004 29902774

[B34] Lu J. Wang Y. Li J. Mao L. Nguyen S. H. Duarte T.. (2018b). Triclosan at environmentally relevant concentrations promotes horizontal transfer of multidrug resistance genes within and across bacterial genera. Environ. Int. 121, 1217–1226. doi: 10.1016/j.envint.2018.10.040 30389380

[B35] Lu J. Wang Y. Zhang S. Bond P. Yuan Z. Guo J. (2020). Triclosan at environmental concentrations can enhance the spread of extracellular antibiotic resistance genes through transformation. Sci. Total Environ. 713, 136621. doi: 10.1016/j.scitotenv.2020.136621 32019018

[B36] Lyte M. (2014). Microbial endocrinology. Gut Microbes 5 (3), 381–389. doi: 10.4161/gmic.28682 24690573 PMC4153777

[B37] Lyte M. Ernst S. (1992). Catecholamine induced growth of gram negative bacteria. Life Sci. 50 (3), 203–212. doi: 10.1016/0024-3205(92)90273-R 1731173

[B38] Lyte M. Frank C. D. Green B. T. (1996). Production of an autoinducer of growth by norepinephrine cultured Escherichia coli O157:H7. FEMS Microbiol. Lett. 139 (2-3), 155–159. doi: 10.1111/j.1574-6968.1996.tb08196.x 8674983

[B39] Lyte M. Villageliú D. N. Crooker B. A. Brown D. R. (2018). Symposium review: Microbial endocrinology—Why the integration of microbes, epithelial cells, and neurochemical signals in the digestive tract matters to ruminant health1. J. Dairy Sci. 101 (6), 5619–5628. doi: 10.3168/jds.2017-13589 29550113

[B40] Maiden M. M. Waters C. M. (2020). Triclosan depletes the membrane potential in Pseudomonas aeruginosa biofilms inhibiting aminoglycoside induced adaptive resistance. PloS Pathog. 16 (10), e1008529. doi: 10.1371/journal.ppat.1008529 33125434 PMC7657502

[B41] Pereira A. R. Gomes I. B. Simões M. (2023). Impact of parabens on drinking water bacteria and their biofilms: The role of exposure time and substrate materials. J. Environ. Manage 332, 117413. doi: 10.1016/j.jenvman.2023.117413 36764214

[B42] Peterson G. Kumar A. Gart E. Narayanan S. (2011). Catecholamines increase conjugative gene transfer between enteric bacteria. Microb. Pathog. 51 (1), 1–8. doi: 10.1016/j.micpath.2011.03.002 21419838

[B43] Qin S. Xiao W. Zhou C. Pu Q. Deng X. Lan L.. (2022). Pseudomonas aeruginosa: pathogenesis, virulence factors, antibiotic resistance, interaction with host, technology advances and emerging therapeutics. Signal Transduct Target Ther. 7 (1), 1–27. doi: 10.1038/s41392-022-01056-1 35752612 PMC9233671

[B44] RAPPORT AST révisé de l’Anses relatif à la Campagne nationale de mesures de l’occurrence de composés émergents dans les eaux destinées à la consommation humaine acides haloacétiques – chlorates – phtalates - Rouxiella chamberiensis, campagne 2016-2017 (2020) (Anses - Agence nationale de sécurité sanitaire de l’alimentation, de l’environnement et du travail). Available at: https://www.anses.fr/fr/content/rapport-ast-r%C3%A9vis%C3%A9-de-lanses-relatif-%C3%A0-la-campagne-nationale-de-mesures-de-l%E2%80%99occurrence-de (Accessed July 10, 2023).

[B45] Rosay T. Bazire A. Diaz S. Clamens T. Blier A. S. Mijouin L.. (2015). Pseudomonas aeruginosa expresses a functional human natriuretic peptide receptor ortholog: involvement in biofilm formation. mBio 6 (4), e01033–e01015. doi: 10.1128/mBio.01033-15 26307165 PMC4550695

[B46] Sakamoto H. Yokota H. Kibe R. Sayama Y. Yuasa A. (2002). Excretion of bisphenol A-glucuronide into the small intestine and deconjugation in the cecum of the rat. Biochim. Biophys. Acta BBA - Gen. Subj 1573 (2), 171–176. doi: 10.1016/S0304-4165(02)00418-X 12399027

[B47] Schug T. T. Janesick A. Blumberg B. Heindel J. J. (2011). Endocrine disrupting chemicals and disease susceptibility. J. Steroid Biochem. Mol. Biol. 127 (3-5), 204–215. doi: 10.1016/j.jsbmb.2011.08.007 21899826 PMC3220783

[B48] Shariati S. Pourbabaee A. A. Alikhani H. A. (2023). Biodegradation of diethyl phthalate and phthalic acid by a new indigenous Pseudomonas putida. Folia Microbiol. (Praha) 68 (3), 477–488. doi: 10.1007/s12223-022-01022-y 36635520

[B49] Shaw C. Hess M. Weimer B. C. (2022). Two-component systems regulate bacterial virulence in response to the host gastrointestinal environment and metabolic cues. Virulence 13 (1), 1666–1680. doi: 10.1080/21505594.2022.2127196 36128741 PMC9518994

[B50] Soni M. G. Carabin I. G. Burdock G. A. (2005). Safety assessment of esters of p-hydroxybenzoic acid (parabens). Food Chem. Toxicol. 43 (7), 985–1015. doi: 10.1016/j.fct.2005.01.020 15833376

[B51] Substituts de phtalates dans les jouets pas de risque mis en évidence pour la santé des enfants de moins de trois ans (2016) (Anses - Agence nationale de sécurité sanitaire de l’alimentation, de l’environnement et du travail). Available at: https://www.anses.fr/fr/content/substituts-de-phtalates-dans-les-jouets-pas-de-risque-mis-en-%C3%A9vidence-pour-la-sant%C3%A9-des (Accessed May 26, 2023).

[B52] Syed A. K. Ghosh S. Love N. G. Boles B. R. (2014). Triclosan promotes staphylococcus aureus nasal colonization. mBio 5 (2), e01015–e01013. doi: 10.1128/mBio.01015-13 PMC3993860 24713325

[B53] Thiroux A. Labanowski J. Venisse N. Crapart S. Boisgrollier C. Linares C.. (2023). Exposure to endocrine disruptors promotes biofilm formation and contributes to increased virulence of Pseudomonas aeruginosa. Environ. Microbiol. Rep. doi: 10.1111/1758-2229.13190 PMC10667657 37586891

[B54] Valcárcel Y. Valdehíta A. Becerra E. López de Alda M. Gil A. Gorga M.. (2018). Determining the presence of chemicals with suspected endocrine activity in drinking water from the Madrid region (Spain) and assessment of their estrogenic, androgenic and thyroidal activities. Chemosphere 201, 388–398. doi: 10.1016/j.chemosphere.2018.02.099 29529566

[B55] Wang Y. Qian H. (2021). Phthalates and their impacts on human health. Healthcare 9 (5), 603. doi: 10.3390/healthcare9050603 34069956 PMC8157593

[B56] Wang H. Yu P. Schwarz C. Zhang B. Huo L. Shi B.. (2022). Phthalate esters released from plastics promote biofilm formation and chlorine resistance. Environ. Sci. Technol. 56 (2), 1081–1090. doi: 10.1021/acs.est.1c04857 34991317

[B57] Weatherly L. M. Gosse J. A. (2017). Triclosan exposure, transformation, and human health effects. J. Toxicol. Environ. Health B Crit. Rev. 20 (8), 447–469. doi: 10.1080/10937404.2017.1399306 29182464 PMC6126357

[B58] Wei F. Mortimer M. Cheng H. Sang N. Guo L. H. (2021). Parabens as chemicals of emerging concern in the environment and humans: A review. Sci. Total Environ. 778, 146150. doi: 10.1016/j.scitotenv.2021.146150 34030374

[B59] Westfall C. Flores-Mireles A. L. Robinson J. I. Lynch A. J. L. Hultgren S. Henderson J. P.. (2019). The widely used antimicrobial triclosan induces high levels of antibiotic tolerance *in vitro* and reduces antibiotic efficacy up to 100-fold *in vivo* . Antimicrob. Agents Chemother. 63 (5), e02312–e02318. doi: 10.1128/AAC.02312-18 30782996 PMC6496070

[B60] Yamazaki E. Yamashita N. Taniyasu S. Lam J. Lam P. K. Moon H. B.. (2015). Bisphenol A and other bisphenol analogues including BPS and BPF in surface water samples from Japan, China, Korea and India. Ecotoxicol Environ. Saf. 122, 565–572. doi: 10.1016/j.ecoenv.2015.09.029 26436777

[B61] Yue B. Ning S. Miao H. Fang C. Li J. Zhang L.. (2023). Human exposure to a mixture of endocrine disruptors and serum levels of thyroid hormones: A cross-sectional study. J. Environ. Sci. 125, 641–649. doi: 10.1016/j.jes.2022.01.017 36375946

[B62] Zhang H. Quan Q. Li X. Sun W. Zhu K. Wang X.. (2020). Occurrence of parabens and their metabolites in the paired urine and blood samples from Chinese university students: Implications on human exposure. Environ. Res. 183, 109288. doi: 10.1016/j.envres.2020.109288 32311914

[B63] Zhu Q. Xu L. Wang W. Liu W. Liao C. Jiang G. (2022). Occurrence, spatial distribution and ecological risk assessment of phthalate esters in water, soil and sediment from Yangtze River Delta, China. Sci. Total Environ. 806, 150966. doi: 10.1016/j.scitotenv.2021.150966 34656589

